# Biobased and Biodegradable Furandicarboxylate Polyesters: Linking Molecular Structure to Enzymatic Hydrolyzability

**DOI:** 10.1021/acs.est.6c01921

**Published:** 2026-06-22

**Authors:** Thijs Vangeel, Yannick Matt, Lukas Becker, Kai Oliver Siegenthaler, Michael Sander

**Affiliations:** † Department of Environmental Systems Science, Institute of Biogeochemistry and Pollutant Dynamics (IBP), ETH Zurich, 8092 Zurich, Switzerland; ‡ Polycondensation Research, RGR/BS, 5184 BASF SE, Carl-Bosch-Strasse 38, Ludwigshafen 67056, Germany; § Biodegradation & Microplastic Research, RGS/AB, BASF SE, Carl-Bosch-Strasse 38, Ludwigshafen 67056, Germany; ∥ Department of Process Engineering, Swiss Federal Institute of Aquatic Science and Technology (Eawag), 8600 Dübendorf, Switzerland

**Keywords:** aliphatic–aromatic copolyester, 2,5-furandicarboxylic acid, biobased biodegradable polyesters, enzymatic hydrolysis

## Abstract

Biobased, biodegradable polyesters are key to advancing a circular polymer economy by ensuring complete microbial utilization of such polyesters in targeted receiving environments. Extracellular enzymatic hydrolysis is often the rate-limiting step in overall polyester biodegradation. This work explores the hydrolytic activity of two esterases, *Humicola insolens* cutinase (HiC) and *Rhizopus oryzae* lipase (RoL), on a large set of 30 aliphatic–aromatic copolyesters. The copolyesters varied systematically in monomer composition, with most containing biobased 2,5-furandicarboxylic acid (F) and, for comparison, some containing fossil-derived terephthalic acid (T). The type and content of the aromatic diacid strongly influenced enzymatic hydrolysis: increasing the aromatic diacid content decreased hydrolysis rates, with F-containing copolyesters being consistently more hydrolyzable than their T analogues at the same aromatic diacid content. Hydrolyzability was further modulated by aliphatic diol and diacid chain lengths, with the combination of a short diol with longer-chain diacids favoring hydrolysis. HiC displayed broad activity across all copolyesters, whereas RoL was active only on (parts of the) polyesters with low aromatic content. By assessing the effect of copolyester chemical structure on polyester enzymatic hydrolyzability, this work informs the design of biobased polyesters with desired biodegradability performance.

## Graphical abstract

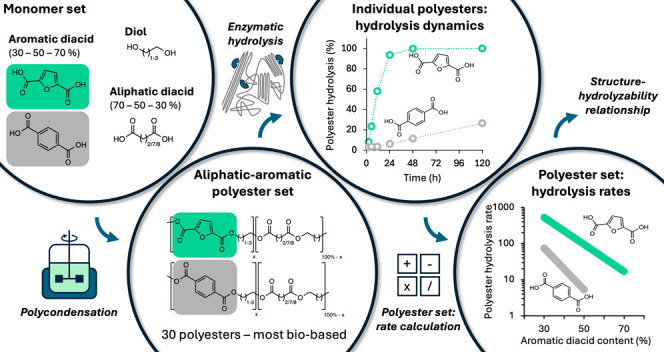

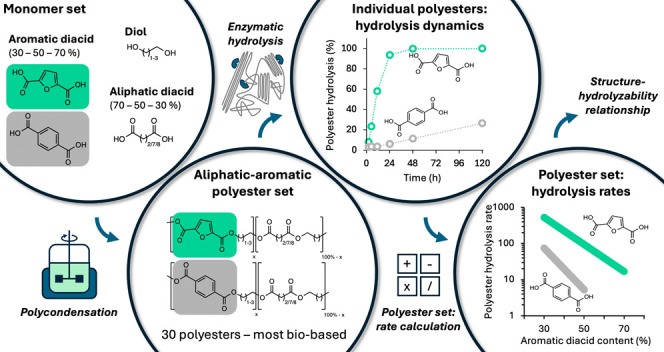

## Introduction

Biodegradable polymers are an integral part of the solution toolbox to achieve a circular plastic economy.^1–3^ Biodegradability ensures that these polymers—following their use in *specific* applications with targeted natural or engineered receiving environments—undergo complete microbial metabolic conversion. By (re)-entering the biological element cycle, biodegradable polymers do not leave behind any material that accumulates, including persistent micro- and nanoplastics.^4,5^ Applications in which the use of biodegradable polymers is advantageous over the use of conventional polymers include those in which the polymer is used in the open environment but cannot be (completely) recollected after use. These include, but are not limited to, agricultural mulch films, plant clips, and geotextiles.^1,6^ Also, there are several applications which have industrial or home compost as targeted systems; these include biodegradable bags to collect household biowaste and biodegradable coffee capsules, as well as certain food packaging, such as polymer-coated paper.

One commercially important class of biodegradable polymers is aliphatic–aromatic copolyesters synthesized from aliphatic and aromatic dicarboxylic acids and aliphatic diols. These polyesters are highly versatile in their physicochemical properties—and thus application domains—due to the modularity of changing the diacids and diols as well as the molar ratio of aliphatic to aromatic diacids.^7,8^ This versatility is, arguably, best documented for poly­(butylene adipate-*co*-terephthalate) (PBAT) polyesters, in which variation of the terephthalic acid-to-total diacid ratio allows tuning material properties as well as enzymatic hydrolyzability and biodegradability.^9–11^ Yet, these biodegradable aliphatic–aromatic copolyesters are still (partly) fossil-based. To alleviate reliance on fossil feedstocks, it is desirable to replace fossil-based monomers with biobased alternatives in polyester synthesis.^12^

Biobased 2,5-furandicarboxylic acid (F)—synthesized from carbohydrates—is an interesting candidate to replace fossil-based terephthalic acid (T) in aliphatic–aromatic polyesters.^13,14^ This replacement promises to become possible at scale given that high-purity monomeric F has recently become available in large quantities.^15^ The replacement of T by F has been explored both in aromatic polyesters (e.g., polyethylene furanoate (PEF) as a substitute for polyethylene terephthalate (PET)) and in selected aliphatic–aromatic copolyesters (e.g., PBAF).^13,15–19^

Irrespective of the receiving environment of the biodegradable material, a critical and often the rate-determining step in polyester biodegradation is the extracellular hydrolytic breakdown to release products of sufficiently low molecular weight to be taken up and metabolized by microorganisms. For aliphatic–aromatic polyesters, ester bond hydrolysis commonly requires catalysis by extracellular esterases secreted by competent fungi and bacteria.^1,20^ Therefore, considerable research efforts have been directed toward demonstrating and understanding enzymatic polyester hydrolysis. These efforts also reflect interest in using enzymatic hydrolyzability (screening) assays to identify polyesters with high biodegradability potential, which subsequently may be selected for costly and time-intensive biodegradation assessment involving incubations coupled to the respirometric analysis of formed polyester-derived CO_2_.^21–25^ In this work, two commercially available esterases—*Humicola insolens* cutinase (HiC) and *Rhizopus oryzae* lipase (RoL)—are used as model enzymes to assess polyester hydrolyzability. These extracellular enzymes are secreted by common soil-dwelling fungi and represent two major esterase classes (cutinases, EC 3.1.1.74; and lipases, EC 3.1.1.3). Given their distinct hydrolytic activities, these enzymes serve as effective proxies for evaluating how chemical structure controls enzymatic hydrolysis.^26–29^ The enzymatic hydrolyzability of aliphatic–aromatic copolyesters depends on their monomeric composition. Initial studies have assessed enzymatic hydrolysis of selected F-containing aliphatic–aromatic copolyesters. First, enzymatic hydrolysis of some copolyesters, including poly­(butylene adipate-*co*-butylene furandicarboxylate) (PBAF), was reported to decrease with increasing F content,^16,30–35^ consistent with effects of T on the enzymatic hydrolysis of PBAT.^8,22,27^ At the same time, enzymatic hydrolyzability^16^ as well as compostability^17^ have been found to be higher for F- than T-containing analogues with comparable aromatic diacid contents. Second, the length of the aliphatic diol was found to affect enzymatic hydrolysis: poly­(alkylene adipate-*co*-furanoate) copolyesters with shorter-chain diols (i.e., 3, 4, and 6 carbon atoms) exhibited faster enzymatic hydrolysis compared to their longer-chain counterparts (i.e., 8, 9, or 10 carbon atoms).^36^ Finally, the type of aliphatic diacid also affected enzymatic hydrolyzability: substituting adipic acid (A in PBAF) with either succinic acid (S in PBSF) or diglycolic acid (D in PBDF) lowered enzymatic hydrolyzability, presumably due to decreasing chain flexibility and thus impairing formation of the enzyme–substrate complex.^37^ Consistently, a lower stability against abiotic hydrolysis and higher biodegradability in industrial compost were reported for PBAF than for PBSF.^32,38^ These initial studies, run under different conditions and with different enzymes and on small polyester sets, call for a comprehensive assessment of an underlying structure–enzymatic hydrolyzability relationship of aliphatic–aromatic F-containing polyesters that vary in the types of aliphatic diacids and diols, as well as aromatic diacid content.

This study presents a systematic assessment of the enzymatic hydrolyzability of a set of structurally diverse, fully biobased aliphatic–aromatic F-based copolyesters by two different extracellular esterases, a cutinase and a lipase. More specifically, we tested a large combinatorial matrix of copolyesters that varied in their diols (i.e., ethylene glycol, 1,3-propanediol, and 1,4-butanediol), their aliphatic diacids (i.e., succinic acid, azelaic acid, and sebacic acid), and their aromatic diacid contents (30%, 50%, and 70% F). Additionally, we compare the enzymatic hydrolyzability of selected F-based polyesters with their T-based counterparts to assess the effects of the type of aromatic diacid on the enzymatic hydrolyzability. The established structure–hydrolyzability relationship will guide the development of F-based aliphatic–aromatic copolyesters with targeted enzymatic hydrolyzability and, likely, biodegradability characteristics.

## Materials and Methods

### Chemicals and Solvents

Chloroform (>99.8%, HPLC grade), 1,4-dinitrobenzene (>98%), sodium dihydrogen phosphate monohydrate (NaH_2_PO_4_·H_2_O), fumaric acid (>99%), sodium hydroxide (>98%), and a total organic carbon (TOC) calibration standard (1000 mg/L) were purchased from Merck. Dichloromethane (>99.8%, HPLC grade) was purchased from Fischer Scientific. Deuterated chloroform (CDCl_3_, 99.8 atom % D) and deuterium oxide (D_2_O, 99.8 atom % D) were purchased from Thermo Scientific. Deuterated dichloromethane (99.5 atom % D) was purchased from Apollo Scientific. All chemicals and solvents were used as received. The monomers used for polymer synthesis are provided in Section S1, Supporting Information.

### Polyesters

A total of 33 aliphatic–aromatic copolyesters, including 27 F-containing and 6 T-containing specimens, were synthesized using the following polycondensation protocol. In brief, for each polyester, the aromatic diacid (F or T) and the respective aliphatic diacid were transferred into a 1 L round-bottom flask—equipped with a stirrer, an inside thermometer, a nitrogen inlet, a heating mantle, and a Dean–Stark apparatus—in ratios indicated in Table 1, all under a nitrogen atmosphere. The respective diol was added, together with 500 ppm tetrabutyl orthotitanate as the catalyst, to achieve a molar ratio of diacids to diol of 1:1.2. The resulting mixture was heated under stirring and nitrogen flow to 190 °C, 210 °C, or 230 °C (for ethylene glycol, 1,3-propanediol or 1,4-butanediol, respectively) until no distillate was formed anymore. Afterward, the nitrogen flow was stopped, temperature was increased to 210 °C, 230 °C, or 240 °C (for ethylene glycol, 1,3-propanediol, or 1,4-butanediol, respectively), and the reaction was placed under vacuum. The reaction mixture was stirred until it reached a torque of 20 N·cm, and the polyester melt was then poured onto a Teflon pad to cool down.

**Table 1:**
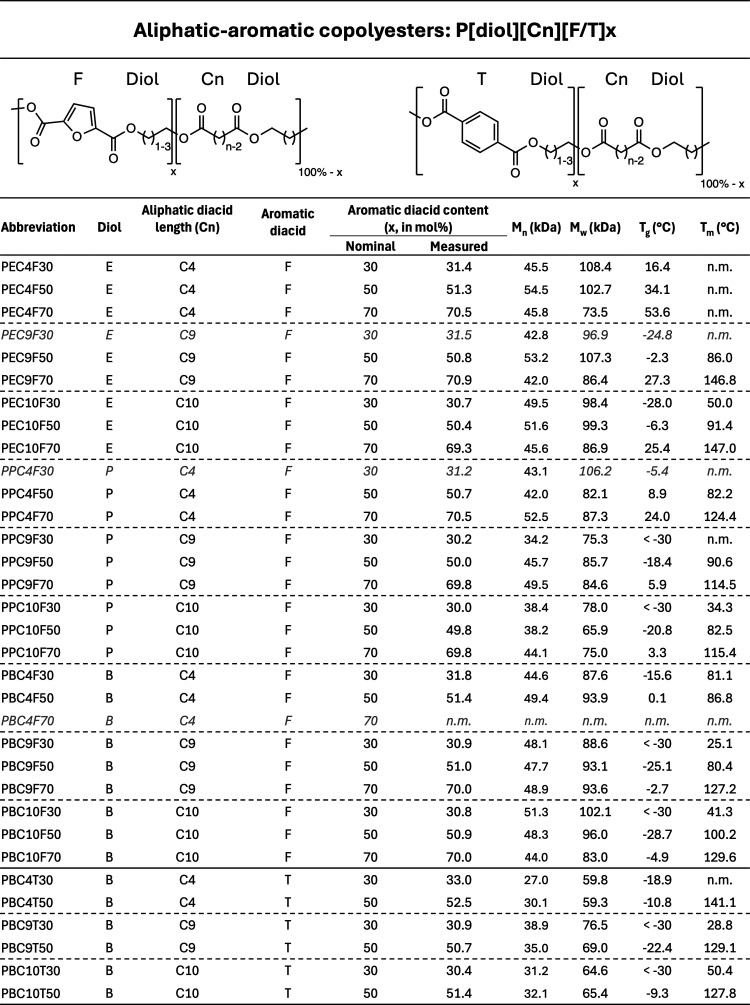
Nomenclature, Chemical Structure, and Key Physicochemical Properties of the Aliphatic–Aromatic Copolyesters Used in This Study^a^

a The general polyester structure is poly­(diol)­(aliphatic diacid)­(aromatic diacid)*x*, where *x* represents the molar ratio of the aromatic diacid relative to the total diacid content: *x* = (F or T)/[(F or T) + (aliphatic diacid)] × 100 (%). The aromatic diacid content that was targeted in synthesis (nominal value) was subsequently confirmed postsynthesis by dissolution of the polyester in deuterated chloroform followed by proton nuclear magnetic resonance (^1^H NMR) analysis. Abbreviations: ethylene glycol (E), 1,3-propanediol (P), 1,4-butanediol (B), 2,5-furandicarboxylic acid (F), terephthalic acid (T), succinic acid (C4), azelaic acid (C9), sebacic acid (C10), number average molecular weight (*M*_n_), mass average molecular weight (*M*_w_), glass transition temperature (*T*_g_), and melting temperature (*T*_m_). n.m. = not measurable. Notes: (i) Polyesters PEC9F30 and PPC4F30 (in gray, italics) were excluded from the enzymatic hydrolysis experiments as these polyesters could not be reproducibly solvent-cast into films. (ii) The synthesis of polyester PBC4F70 (in gray, italics) was unsuccessful, as no significant increase in *M*_w_ could be achieved. (iii) *T*_g_ measurements were possible only above −30 °C such that values below −30 °C cannot be reported correctly.

All polyesters were characterized by differential scanning calorimetry (DSC, TA Instruments Q2000; heating rate 20 K·min^–1^) to assess their glass transition temperature (*T*_g_) and melting point (*T*_m_). Thermal properties were determined from the first heating scan for all polyester samples, except for PBC9F30, PBC10F30, and PPC10F30, as these polyesters exhibited melting temperatures below the DSC standby temperature (38 °C). The first heating scan was used for analysis as these polyesters show only slow crystallization kinetics. Gel permeation chromatography (GPC) of the synthesized polyesters was carried out in hexafluoroisopropanol containing 0.05% potassium trifluoroacetate, calibrated to a poly­(methyl methacrylate) standard, to determine the polyester number and mass average molecular weight (*M*_n_ and *M*_w_).

The polyesters varied in their molar ratio of aromatic (F or T) to total (i.e., aromatic + aliphatic) diacid (hereafter referred to as *x* = 30, 50, and 70%), the length of the aliphatic diacid (i.e., succinic acid (C4), azelaic acid (C9), and sebacic acid (C10)), and the length of the diol (i.e., ethylene glycol (E), 1,3-propanediol (P), and 1,4-butanediol (B)). The chemical structures, corresponding nomenclatures, and key physicochemical properties of the tested polyesters are summarized in Table 1.

### Polyester Characterization by ^1^H NMR

Most polyesters were well-soluble in deuterated chloroform for analysis, except for PEC4F70, PEC9F70, and PEC10F70, which had to be dissolved in deuterated dichloromethane. Both solvents contained known amounts of 1,4-dinitrobenzene as an internal quantitation standard. ^1^H NMR spectra were acquired using a Bruker Avance III 400 MHz NMR spectrometer equipped with a 5 mm BBFO Z-Gradient high-resolution probe. For each sample, 16 dummy scans and 64 measurement scans were acquired, with a 14 μs pulse width and a 15 s delay time between scans. The polyester aromatic diacid contents were calculated based on the integrals of the signals from the diol’s outer methylene protons next to the aromatic and the aliphatic diacids (Table 1; corresponding spectra are shown in Section S2).

### Preparation of Polyester Films

Films were prepared by solvent casting. To this end, polyesters were dissolved in chloroform—except PEC4F70, PEC9F70, and PEC10F70, which were dissolved in dichloromethane—to obtain 4% (w/v) polyester solutions. Films were cast by pipetting 541 μL of the respective polyester solution onto a circular polypropylene dish (diameter: 2.7 cm), followed by letting the chloroform or dichloromethane evaporate at room temperature. To evaluate the impact of crystallinity on enzymatic hydrolyzability, we assessed the effect of thermal annealing on the hydrolyzability of PBC9F30, PBC9F50, and PBC9F70. Following standard solvent-casting of these films, a subset was thermally annealed at 70 °C for 2 h within the casting dishes, with the complementary subset being stored at room temperature only. The resulting circular films were peeled from the individual dishes, which, for polyesters PPC9F30, PPC10F30, and PEC9F50, required brief cooling of the dishes at −20 °C. The resulting films had an approximate thickness of 30 μm, weighed approximately 21 mg—with the exact mass of each film being gravimetrically determined and used in data evaluation—and had similar surface areas, ensuring comparability of hydrolysis data across films. PEC9F30 and PPC4F30 had to be omitted from the enzymatic hydrolysis experiments given that they were very sticky, making it impossible to obtain well-formed cast films.

### Esterases

Cutinase from *H. insolens* (HiC; 20.25 kDa) was obtained from ChiralVision (StickAway, product number NZ51032, Novozymes) as a solution (12 g/L active enzyme protein, stabilized with glycerol and sodium chloride). The activity of this lot was 28,000 TBU/mL (where 1 TBU = 1 μmol butyric acid released·min^–1^ at pH 7.5 at 40 °C in the hydrolysis of 1% tributyrin). The enzyme solution was aliquoted, stored at −20 °C, and used as received in hydrolysis experiments.

Lipase from *R. oryzae* (RoL; 29.6 kDa) was obtained from Sigma-Aldrich (product number 80612; lot activity of 35.2 U/mg (where 1 U = 1 μmol fatty acid released·min^–1^ at pH 7.2 at 37 °C in the hydrolysis of olive oil)), stored at −20 °C, and used as received in hydrolysis experiments.

### Enzymatic Hydrolysis Experiments

Enzymatic hydrolysis experiments were carried out in triplicate on films for each polyester specimen and enzyme combination. For each experiment, a circular polyester film was transferred into a 20 mL glass vial with Teflon-lined screw cap. To start the experiment, 20 mL of a phosphate-buffered solution containing either HiC or RoL was added to the vial. The phosphate buffer (100 mM) was prepared using sodium dihydrogen phosphate monohydrate (NaH_2_PO_4_·H_2_O) and adjusted to pH 7.1 using a sodium hydroxide (NaOH) solution. Buffering served to avoid solution acidification from proton release associated with ester bond hydrolysis. The final enzyme concentrations were 5.12 mg L^–1^ (0.253 μM) for HiC and 177.6 mg L^–1^ (6 μM) for RoL, with the lower HiC concentration reflecting its substantially higher hydrolytic activity on the polyesters compared to RoL. The enzymatic hydrolysis was carried out at 25 °C while horizontally orbital-shaking the vials at 150 rpm.

Throughout the experiment, 0.75 mL aliquots were periodically withdrawn from each vial and filtered through 0.22 μm pore size syringe filters (PVDF membranes, BGB) to remove potentially present nondissolved film particles. The filtrate was frozen to later allow for batch processing of multiple samples by total organic carbon (TOC) analysis. Control experiments were run containing enzyme solution but no polyester films to correct for background TOC originating from the enzyme addition.

For TOC measurement, each frozen sample was thawed, and a 0.5 mL aliquot was diluted with 7.5 mL ultrapure water (Milli-Q IQ 7000 system, resistivity 18.2 MΩ·cm) in a glass test tube, mixed thoroughly, and then analyzed for nonpurgeable organic carbon (NPOC) using a TOC analyzer (TOC-L, Shimadzu). The method included acidification (addition of 1.5% of 2 M HCl) and sparging (80 mL air/min for 1.5 min) to liberate and remove CO_2_ from potentially present carbonates. The measured NPOC value was converted to a TOC concentration employing a TOC standard calibration curve (0 to 50 mg C L^–1^) prepared by dilution from a TOC standard solution (1000 mg C L^–1^).

Next, a subset of polyester–enzyme combinations was examined in more detail for product analysis by ^1^H NMR: RoL with PBC9F30 and HiC with PBC9F30, PBC9F50, and PBC9F70. To this end, additional enzymatic hydrolysis experiments were carried out, using identical conditions to those described above. After a defined reaction time, the entire sample was filtered through a Por. 4 glass filter (pore size: 10–16 μm). From the filtrate, 4 mL was freeze-dried and reconstituted in deuterium oxide (D_2_O) containing fumaric acid as an internal standard for ^1^H NMR analysis. Enzyme solutions without polyester were processed and analyzed in the same way to serve as controls.

The residual polyester particulates retained on the glass filter were washed with ultrapure water. The remaining particulates were then dissolved with 5 mL of dichloromethane and passed through the same glass filter. This was repeated three times to ensure the complete dissolution of the particulates on the filter. The dichloromethane fractions were then combined and evaporated to dryness, and the dried residue was subsequently reconstituted in CDCl_3_ with 1,4-dinitrobenzene as an internal standard for ^1^H NMR analysis.

Both filtrate and retentate samples (in D_2_O and CDCl_3_, respectively) were analyzed by ^1^H NMR using the same acquisition settings as above for pure polyesters. The amount of residual polyester particulates was quantified from the internal standard signal (i.e., 1,4-dinitrobenzene) and the diol outer methylene proton signals. Aromatic diacid content was calculated from diol outer methylene signals for the residual solid polyester in the retentate and from the furan ring proton signals of F for the hydrolysis products in the filtrate.

## Results and Discussion

### Polyester Hydrolysis by *H. insolens* Cutinase

Figure 1 provides an overview of the *H. insolens* cutinase (HiC)-catalyzed hydrolysis experiments of polyesters containing 2,5-furandicarboxylic acid (F) (panel A) and, for comparison, terephthalic acid (T) (panel B). The graphs in a column show polyesters with the same diol (i.e., either ethylene glycol (E), 1,3-propanediol (P), or 1,4-butanediol (B)), and the graphs in a row show polyesters with the same aliphatic diacid (i.e., succinic acid (C4), azelaic acid (C9), or sebacic acid (C10)). Each graph depicts the enzymatic hydrolysis dynamics of polyesters that have the same monomeric composition but vary in the molar ratio of the aromatic diacid relative to the total diacid content (i.e., *x* = 30%, 50%, or 70%).

**Figure 1:**
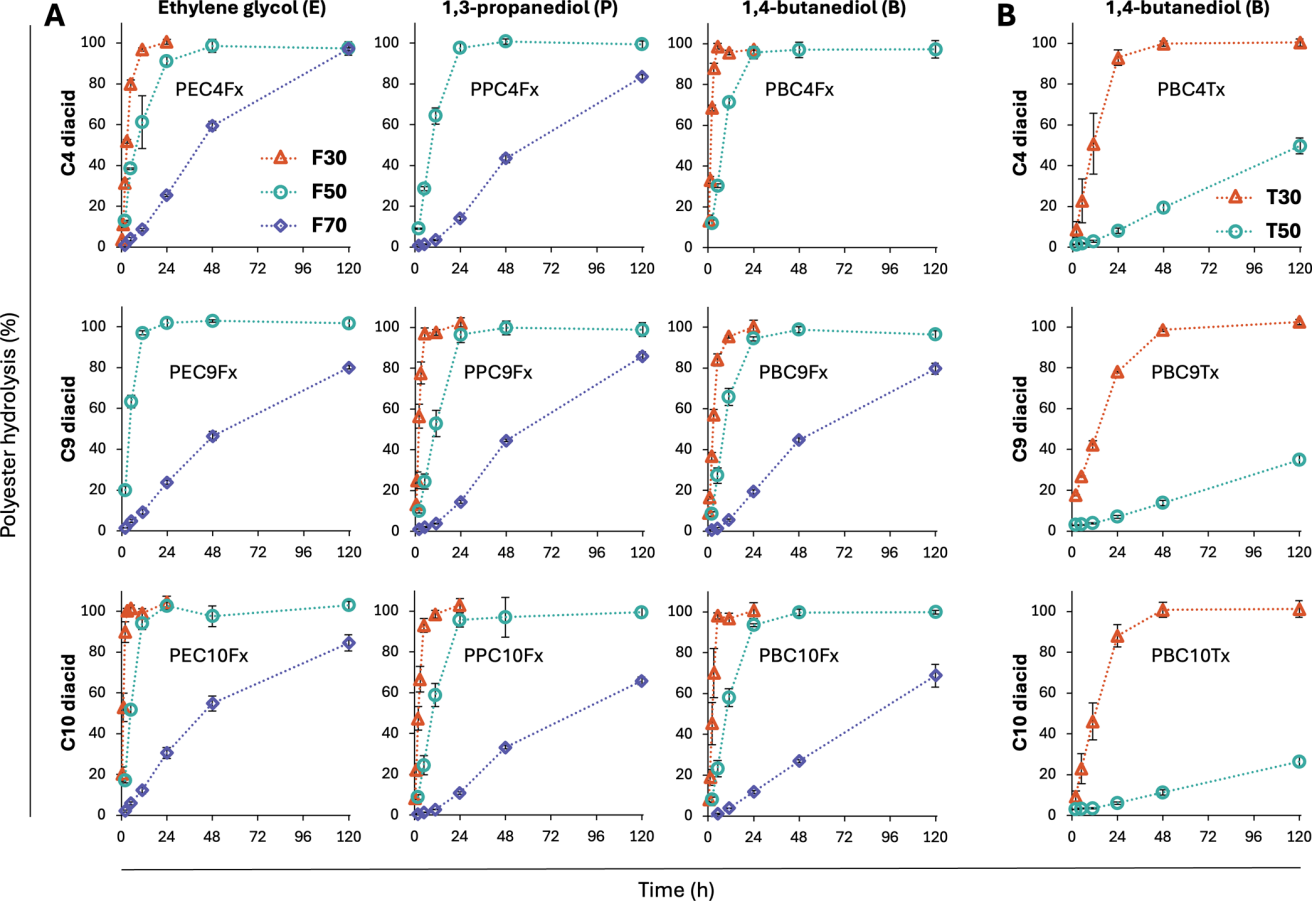
Enzymatic hydrolysis of polyester films by *H. insolens* cutinase (HiC) at pH 7.1 and 25 °C. Small products released during hydrolysis from polyester films into solution were quantified by dissolved total organic carbon (TOC) analysis. The polyester hydrolysis extent is expressed as TOC in solution relative to the amount of carbon initially added in the polyester film (with 100% corresponding to complete transformation of the added polyester carbon into dissolved products). The general polyester structure is P­(diol)­(aliphatic diacid)­(aromatic diacid)*x*, where *x* represents the molar ratio of the aromatic diacid relative to total diacids (i.e., *x* = (F or T)/[(F or T) + (aliphatic diacid)] × 100 (%)). The columns show polyesters with the same diol but varying aliphatic diacids, while the rows represent polyesters with the same aliphatic diacid but varying in the diols. The tested diols were ethylene glycol (E), 1,3-propanediol (P), and 1,4-butanediol (B), and the aliphatic diacids were succinic acid (C4), azelaic acid (C9), and sebacic acid (C10). (A) Polyesters containing 2,5-furandicarboxylic acid, F, as the aromatic diacid. (B) Polyesters containing terephthalic acid, T, as the aromatic diacid. Polyesters with different aromatic contents (i.e., sets of F30, F50, and F70 and sets of T30 and T50) are shown in different colors. Note that polyesters PEC9F30, PPC4F30, and PBC4F70 were excluded from enzymatic hydrolysis due to poor film-forming ability and/or inadequate material properties. Symbols and error bars represent means and corresponding standard deviations of triplicate hydrolysis experiments for each polyester.

The total organic carbon (TOC) in solution during enzyme incubation increased over time across all tested polyesters (Figure 1). The highest measured TOC values corresponded to about 100% of the carbon added as polyester films, implying that they were completely converted into water-soluble products (i.e., “dissolved organic matter” operationally defined by passing through a <0.22 μm cutoff membrane filter). Error bars capturing standard deviations between triplicate measurements were generally small, highlighting both the reproducibility of the experimental approach and the reliability of the analytical process.

Figure 1 shows two major effects of the polyester structure on hydrolyzability. First, hydrolysis by HiC markedly slowed down as the content of aromatic diacid increased from 30 to 50 and 70%. This trend holds true for both F-containing (Figure 1A) and T-containing (Figure 1B) polyesters. For example, after 11 h of incubation, hydrolysis was (close to) complete for all tested polyesters with 30% F, decreased to between 53 and 97% for polyesters containing 50% F, and was less than 13% for those containing 70% F. Similarly, for T-containing polyesters, hydrolysis after 48 h was complete for the 30% T variants but remained incomplete (extents between 11 and 20%) for the 50% T variants. The consistent trend of decreasing enzymatic hydrolyzability with increasing F or T content across a wide range of polyesters extends previous findings for F- and T-based polyesters.^16,22,27,34,35^

Second, HiC-catalyzed hydrolysis of F-containing polyesters (Figure 1A) was much faster than that of the corresponding T-containing polyesters with the same aromatic diacid content (Figure 1B). For example, F50 variants underwent complete hydrolysis by HiC within 48 h, whereas hydrolysis extents of T50 variants remained below 50% even after 5 days. Therefore, replacing T with F while maintaining the same aromatic content increases polyester enzymatic hydrolyzability (and, thus, likely also biodegradability). This effect was previously reported only for the pair of PBA**F**50 and PBA**T**50.^16^

To enable a quantitative comparison of the hydrolyzability of different polyesters by HiC, the hydrolysis rate (average of triplicates) was determined based on the time required to achieve a hydrolysis extent of 50%. This time was estimated by linear fitting of the initial hydrolysis data (and for selected T-containing polyesters, it required extrapolation). Rates are expressed as the amount of polyester hydrolyzed over a given period, normalized to the surface area of the polyester films (units: μg polyester cm^–2^ h^–1^).

Figure 2 shows the hydrolysis rates of the tested polyesters by HiC, highlighting the effect of the aromatic diacid (Figure 2A) as well as that of the diol and the aliphatic diacid (Figure 2B). As described qualitatively above, the hydrolysis rates decreased substantially with increasing aromatic diacid (F or T) content (Figure 2A). For example, increasing the F content from 30% to 50% resulted in an approximately 4-fold average decrease in the hydrolysis rate, while increasing the F content from 50 to 70% led to an even larger average 8-fold decrease. Identical percentage point increases in the F contents can thus have different effects on the enzymatic hydrolyzability, depending on the absolute range of the F content. For T-containing polyesters, the effect of increasing the aromatic diacid content is even more pronounced: increasing the T content from 30% to 50% resulted in an approximately 14-fold decrease in the average hydrolysis rate. We note that polyesters with 70% T were not synthesized due to an expected very low enzymatic hydrolyzability. Additionally, Figure 2A shows that replacing terephthalic acid (T) with 2,5-furandicarboxylic acid (F) increased hydrolyzability by HiC approximately 6-fold (at 30% F/T) to 19-fold (at 50% F/T) on average.

**Figure 2:**
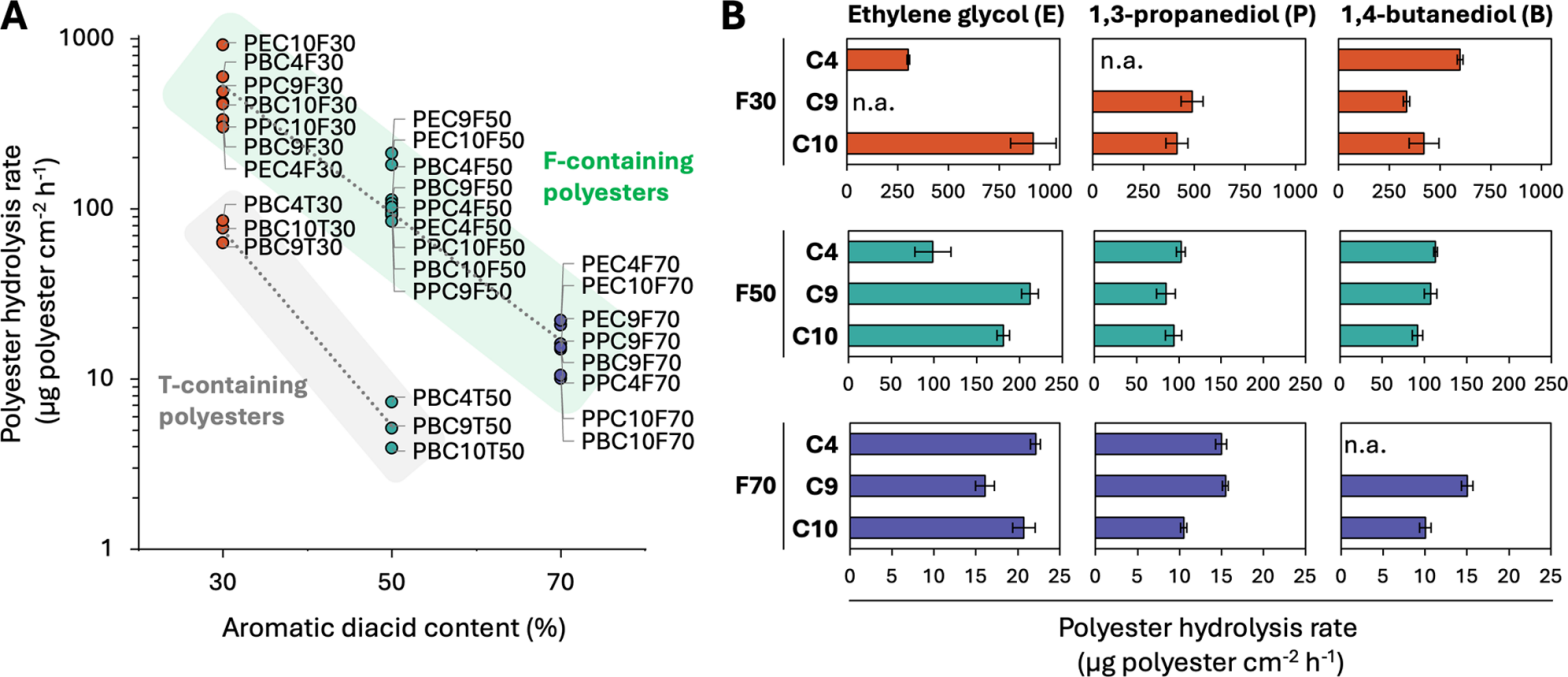
Rates of hydrolysis of polyester films by *H. insolens* cutinase (HiC), calculated as the average surface-area-normalized hydrolysis rate up to hydrolysis extents of 50%. Hydrolysis rates are expressed as the mass of polyester hydrolyzed per polyester film surface area and time (units of μg polyester cm^–2^ h^–1^). (A) Average hydrolysis rate as a function of aromatic diacid content, illustrating the dominant effects of both the content and type of aromatic diacid (2,5-furandicarboxylic acid (F) vs terephthalic acid (T)) on polyester hydrolyzability by HiC. (B) Enzymatic hydrolysis rates of F-containing polyesters. The polyesters are grouped by aromatic diacid content in three rows, emphasizing the effects of diol and aliphatic diacid structure on enzymatic hydrolyzability. Note the change of *x*-axis scales between rows. The diol (i.e., ethylene glycol (E), 1,3-propanediol (P), and 1,4-butanediol (B)) varies horizontally across the panels, and the aliphatic diacid (i.e., succinic acid (C4), azelaic acid (C9), and sebacic acid (C10)) varies vertically within each graph. Aromatic diacid content is denoted as F*x* or T*x*, where *x* represents the molar ratio of the aromatic diacid relative to the total diacid content: *x* = (F or T)/[ (F or T) + (aliphatic diacid)] × 100 (%).

Besides the comparatively large dependence on the content and type of aromatic diacids, the hydrolysis rate of the polyesters was further modulated by the aliphatic diacid and the diol (Figure 2B; note the different *x*-axis scales for the panels in different rows).

Polyesters containing long-chain aliphatic diacids (i.e., C9 or C10) showed faster hydrolysis by HiC when the diol chain length was the shortest (i.e., ethylene glycol). For example, P**E**C10F30 underwent hydrolysis at a rate more than twice the rates of P**P**C10F30 and P**B**C10F30. Also at 50% F, the hydrolysis rate was approximately twice as high for P**E**C9F50 and P**E**C10F50 as that for their counterparts with 1,3-propanediol (P) or 1,4-butanediol (B).

Contrarily, for polyesters containing a short-chain aliphatic diacid (i.e., succinic acid, C4), the opposite trend was observed at 30–50% F: hydrolysis rates were highest for the longest diols (i.e., 1,4-butanediol). For example, P**B**C4F30 underwent hydrolysis at a rate approximately twice that of its counterpart with ethylene glycol P**E**C4F30. At 70% F (and thus 30% C4 diacid content), we observed a reversal of this trend with faster hydrolysis for P**E**C4F70 than for P**P**C4F70.

Overall, the data suggest that at low F contents (30–50%), combinations of a short diol and a long aliphatic diacid (i.e., ethylene glycol and a C9 or C10 diacid) or a long diol and a short aliphatic diacid (i.e., 1,4-butanediol and a C4 diacid) are favorable for enzymatic hydrolysis. At a high F content (70%), the effect of F is dominant, and hydrolysis is fastest with ethylene glycol as the diol.

### Polyester Hydrolysis by *R. oryzae* Lipase

To assess the esterase specificity of the hydrolysis of F-containing polyesters, we repeated the experiments with *R. oryzae* lipase (RoL) at higher enzyme concentrations but under otherwise identical conditions. Hydrolysis was monitored again by quantifying the total organic carbon (TOC) in the solution. Figure 3 provides an overview of the enzymatic hydrolysis of polyesters by RoL.

**Figure 3:**
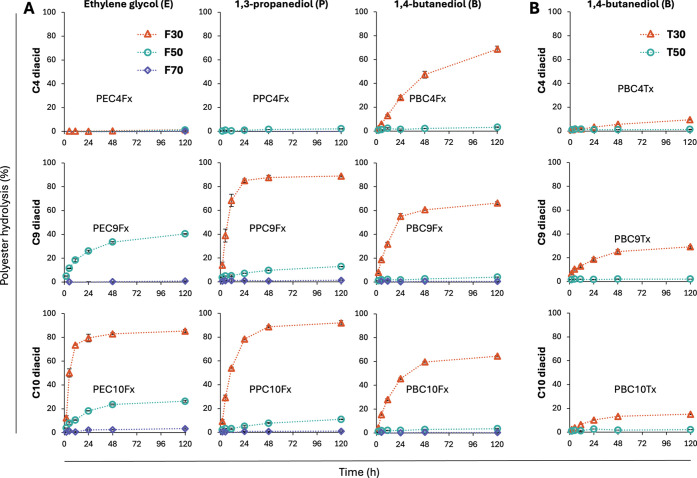
Enzymatic hydrolysis of polyester films by *R. oryzae* lipase (RoL) at pH 7.1 and 25 °C. Small products released during hydrolysis from polyester films into solution were quantified by dissolved total organic carbon (TOC) analysis. The polyester hydrolysis extent is expressed as TOC in solution relative to the amount of carbon initially added in the polyester film (with 100% corresponding to complete transformation of the added polyester carbon into dissolved products). The general polyester structure is P­(diol)­(aliphatic diacid)­(aromatic diacid)*x*, where *x* represents the molar ratio of the aromatic diacid relative to total diacids (i.e., *x* = (F or T)/[(F or T) + (aliphatic diacid)] × 100 (%)). The columns show polyesters with the same diol but varying aliphatic diacids, while the rows represent polyesters with the same aliphatic diacid but varying in the diols. The tested diols were ethylene glycol (E), 1,3-propanediol (P), and 1,4-butanediol (B), and the aliphatic diacids were succinic acid (C4), azelaic acid (C9), and sebacic acid (C10). (A) Polyesters containing 2,5-furandicarboxylic acid, F, as the aromatic diacid. (B) Polyesters containing terephthalic acid, T, as the aromatic diacid. Polyesters with different aromatic contents (i.e., sets of F30, F50, and F70 and sets of T30 and T50) are shown in different colors. Note that polyesters PEC9F30, PPC4F30, and PBC4F70 were excluded from enzymatic hydrolysis due to poor film-forming ability and/or inadequate material properties. Symbols and error bars represent means and corresponding standard deviations of triplicate hydrolysis experiments for each polyester.

Comparing Figures 1 and 3 reveals a considerably lower activity of RoL than HiC on the polyesters: RoL effectively hydrolyzed (i.e., extents >50%) only polyesters with 30% F within 5 days, as opposed to HiC, which completely hydrolyzed polyesters containing 30 and 50% F and extensively hydrolyzed even those with 70% F over the same time. Furthermore, unlike for HiC, RoL-catalyzed polyester hydrolysis extents often plateaued well before reaching complete hydrolysis of the film into dissolved carbon. For instance, PEC10**F30** reached a maximum hydrolysis extent of ∼85%, whereas PEC10**F50** plateaued at ∼26%. Such incomplete hydrolysis is consistent with earlier reports of plateauing hydrolysis by RoL at intermediate extents for PBAT containing 29% and 42% T.^27^

Although RoL exhibited lower overall hydrolytic activity than HiC, key trends observed with HiC were also observed for RoL. First, increasing the aromatic diacid content—for both F and T—lowered hydrolyzability. Most polyesters with 30% F were still hydrolyzed by RoL, but hydrolysis decreased sharply at 50% F and was nearly absent at 70% F. A similar trend was observed for T-containing polyesters: polyesters with 30% T showed some hydrolyzability by RoL (9–29%), whereas those with 50% T were barely hydrolyzed. This is in agreement with literature on PBAT*x*: hydrolysis by RoL was found to be highly dependent on T content, with hydrolysis extent substantially decreasing from 20% T (almost complete hydrolysis) to 50% T (almost no hydrolysis).^27^

Second, F-containing polyesters were more hydrolyzable by RoL than their T-containing counterparts with the same percentage of aromatic diacid. For example, polyesters containing 30% T only reached a final hydrolysis extent of 9–29%, whereas the corresponding F-containing polyesters attained 64–69%.

Third, the diol and aliphatic diacid further modulated hydrolyzability by RoL, consistent with trends seen for HiC. Polyesters with long-chain aliphatic diacids (i.e., C9 or C10) exhibited the highest hydrolyzability when combined with the shortest diol (i.e., ethylene glycol). Conversely, for polyesters containing a short-chain aliphatic diacid (i.e., C4), increasing the diol length markedly increased hydrolyzability: P**E**C4F30 was not hydrolyzed by RoL, whereas P**B**C4F30 underwent extensive hydrolysis (>60%).

In addition to the lower hydrolytic activity of RoL, other differences were observed when comparing RoL and HiC. First, RoL-catalyzed hydrolysis of polyesters containing a C4 aliphatic diacid was consistently slower than that of their C9 or C10 counterparts. This was not observed for HiC-catalyzed hydrolysis. Second, PEC4F30 was particularly resistant to RoL hydrolysis: it remained intact whereas, all other polyesters containing 30% F or T showed some degree of hydrolysis. By comparison, HiC readily hydrolyzed PEC4F30 and did so more efficiently than all other polyesters containing 30% T or 50% F. Third, RoL-catalyzed hydrolysis was faster for polyesters with 30% T than for the corresponding polyesters with 50% F (having the same diol and aliphatic diacid). The opposite was observed for HiC.

### Mechanistic Interpretation

The above results show that the enzymatic hydrolysis of F- (and T-) containing copolyesters is dependent on both polyester and esterase characteristics. This section aims to provide a mechanistic interpretation of the observed dependencies.

#### Polyester Factors

The pronounced decrease in enzymatic hydrolyzability observed for both HiC and RoL with increasing aromatic diacid content can be rationalized by its impact on polyester morphology. Aromatic diacids promote strong intermolecular interactions, notably π–π stacking between aromatic rings on neighboring polymer chains. These interactions favor chain alignment and the formation of crystalline lamellae. Such crystalline domains are likely enriched in aromatic diacid–diol sequences, resulting in an effectively higher aromatic content than that of the bulk polymer.

Most polyesters investigated in this study are semicrystalline, as evidenced by the presence of a melting temperature (*T*_m_)—the temperature at which crystalline domains melt—measured by DSC (Table 1). Furthermore, *T*_m_ increased systematically with increasing aromatic diacid content, consistent with stronger chain–chain interactions within crystalline lamellae driven by π–π stacking between aromatic rings. The low chain mobility within crystalline regions hampers the formation of enzyme–substrate complexes, as polymer chains must partially extend into the enzyme active site for hydrolysis to occur. Consequently, increased intermolecular interactions, higher *T*_m_ values, and reduced chain mobility can explain the observed decrease in enzymatic hydrolysis rates (and, for RoL, also hydrolysis extents) with increasing aromatic diacid content.^21,23,39,40^

This explanation is supported by a negative correlation between HiC-catalyzed hydrolysis rates and polyester *T*_m_, as well as between the extent of RoL-catalyzed hydrolysis after 120 h and *T*_m_ (Section S3). However, these correlations are not very strong, and also no significant correlation was observed between hydrolysis rates and polyester *T*_g_ (Section S3). In fact, stronger correlations were observed between HiC hydrolysis rates and polyester F content than with *T*_m_ (Section S3), consistent with previous reports showing that hydrolysis rates of PBAT*x* polyesters by *Fusarium solani* cutinase correlate more strongly with T content than with *T*_m_.^27^ These observations suggest that the aromatic diacid content captures additional structural effects beyond *T*_m_ alone.

We expect that correlations would improve if enzymatic hydrolysis were related not only to *T*_m_ but also to polyester crystallinity, i.e., the volume fraction of crystalline domains. However, absolute crystallinity could not be determined herein due to unknown enthalpies of fusion of the studied polyesters. To assess the effect of crystallinity, we conducted control experiments on the series PBC9F30, PBC9F50, and PBC9F70 by comparing solvent-cast films with those subjected to thermal annealing (70 °C, 2 h) after solvent casting and prior to enzymatic hydrolysis (Section S3). While annealing and the resulting increase in film crystallinity lowered enzymatic hydrolysis rates, the effect of annealing was much smaller than the effect of changes in the polyester aromatic diacid content on hydrolysis. Future enzymatic hydrolysis studies of F-containing polyesters with known enthalpies of fusion could further assess if improved correlations are obtained when including not only *T*_m_ but also crystallinity characteristics. Polyester morphology also provides a rationale for the substantially higher hydrolyzability of F-containing polyesters compared to their T-containing counterparts at identical aromatic diacid contents. In crystalline domains, furandicarboxylate units are known to pack less efficiently than terephthalate units. The furan ring is smaller and asymmetric, introducing slight kinks and a nonlinear backbone geometry that disrupts close packing.^41–43^ This reduced packing efficiency is reflected in the lower *T*_m_ values of F-containing polyesters compared to T-containing analogues at 50 mol % aromatic diacid content (e.g., *T*_m_ = 80.4 °C for PBC9**F**50 compared to 129.1 °C for PBC9**T**50). In addition, studies on PEF and PET suggest that F-containing polyesters crystallize more slowly than T-containing ones, which may lead to lower overall crystallinity and, consequently, enhanced enzymatic hydrolyzability.^41,44,45^ This may explain why, at 30 mol % aromatic diacid content, F-containing polyesters hydrolyzed faster than their T-containing analogues despite similar *T*_m_ values (e.g., 25.1 °C for PBC9F30 compared to 28.8 °C for PBC9T30). It is also possible that, at lower aromatic diacid contents, crystalline domains are dominated by aliphatic diacid–diol sequences rather than aromatic ones, a phenomenon previously reported for PBATx copolyesters.^9^

Beyond aromatic content, enzymatic hydrolyzability was further modulated by the specific combination of aliphatic diacid and diol. At F contents of 30 and 50 mol %, hydrolyzability was generally enhanced either by pairing long-chain aliphatic diacids (C9 or C10) with a short diol (ethylene glycol) or by pairing a short aliphatic diacid (C4) with a longer diol (e.g., 1,4-butanediol). Polyesters composed of both long aliphatic diacids and long diols may favor chain alignment and increased London dispersion forces, thereby reducing chain mobility and hindering enzyme–substrate complex formation. Conversely, polyesters composed of both short aliphatic diacids and short diols may exhibit stronger dipole–dipole interactions, similarly restricting chain mobility and lowering hydrolyzability.

In addition to chain mobility, surface properties may contribute to these trends. Increased surface apolarity may promote esterase binding and activation—particularly for RoL—whereas more polar polyester surfaces, typical of short diol–short diacid combinations, may impair enzyme activation. Ester bond density at the surface may also influence hydrolysis by determining the number of accessible cleavage sites, potentially slowing hydrolysis in polyesters composed of both long diacids and long diols. The relative contributions of chain mobility, surface polarity, and ester bond density remain to be resolved and warrant dedicated follow-up studies.

#### Enzyme Factors

The differences in the hydrolytic activities of HiC and RoL can be rationalized based on their active site architecture. HiC, as well as many cutinases, has a relatively open, accessible active site and, therefore, a broader substrate specificity, including polyesters with low chain mobility (e.g., highly crystalline polyesters with high *T*_m_).^23,24,39,46,47^ In fact, many enzymes capable of depolymerizing poly­(ethylene terephthalate) are cutinases, and enzyme engineering to further expose the active site has been shown to increase activity.^48,49^

The active site of RoL, as for other lipases, is located within a deeper substrate-binding groove with a lid-like structure covering the active site. Access to the groove and active site requires displacement of this lid through interaction with an apolar surface, a process known as “interfacial activation”. Following lid opening, the polyester chain must extend into the groove to form the enzyme–substrate complex, which in turn requires sufficient chain mobility. As a result, lipases typically exhibit narrower substrate specificity and lower hydrolytic activity than cutinases on polyesters with restricted chain mobility.^23,24,27,39,46^ This likely explains why substantial hydrolysis by RoL was observed only for polyesters containing 30% F. The requirement for interfacial activation may also explain why RoL-catalyzed hydrolysis of polyesters containing a C4 aliphatic diacid was slower than that of polyesters containing C9 or C10 aliphatic diacids. The short C4 segment renders the polyester surface comparatively polar, which may impair lid opening and thereby limit access to the active site. Among the polyesters containing 30 mol % F, PEC4F30 was particularly resistant to RoL-catalyzed hydrolysis: the combination of the C4 diacid with the shortest diol, ethylene glycol, likely resulted in a polyester surface that is insufficiently apolar to effectively trigger interfacial activation.

The interplay between polyester morphology and esterase active site geometry is particularly evident from the observation that RoL-catalyzed hydrolysis often plateaued at intermediate extents. Following the above explanation, this behavior is consistent with preferential hydrolysis of the—presumably amorphous—domains of the polyester with high polyester chain mobility, leading to progressive enrichment of—presumably crystalline—domains with lower chain mobility that are poorly hydrolyzable by RoL. To provide independent support for this interpretation, additional enzymatic hydrolysis experiments were conducted in which both the residual solid polyester and the released, dissolved hydrolysis products were analyzed by ^1^H NMR over the course of the reaction.

During HiC-catalyzed hydrolysis of PBC9F30, PBC9F50, and PBC9F70, the F content of both the residual polyester and the dissolved products remained comparable to that of the initially added bulk polyesters throughout the reaction (Figure 4). This absence of pronounced selectivity indicates that HiC was active on all parts of the polyester. By contrast, RoL-catalyzed hydrolysis of PBC9F30 resulted in a substantial increase in the F content of the residual polyester, from 31% initially to 44% at a hydrolysis extent of 77% (Figure 4). Consistently, the dissolved hydrolysis products were depleted in F relative to the initial bulk polyester. RoL therefore selectively cleaved aliphatic-enriched and F-depleted—presumably amorphous—domains, resulting in a relative enrichment of F-rich—presumably crystalline—domains in the residual solid. In line with this interpretation, negligible hydrolysis by RoL was observed for PBC9F50, whose initial F content (50%) already exceeds the 44% F reached in PBC9F30 at its hydrolysis plateau.

**Figure 4:**
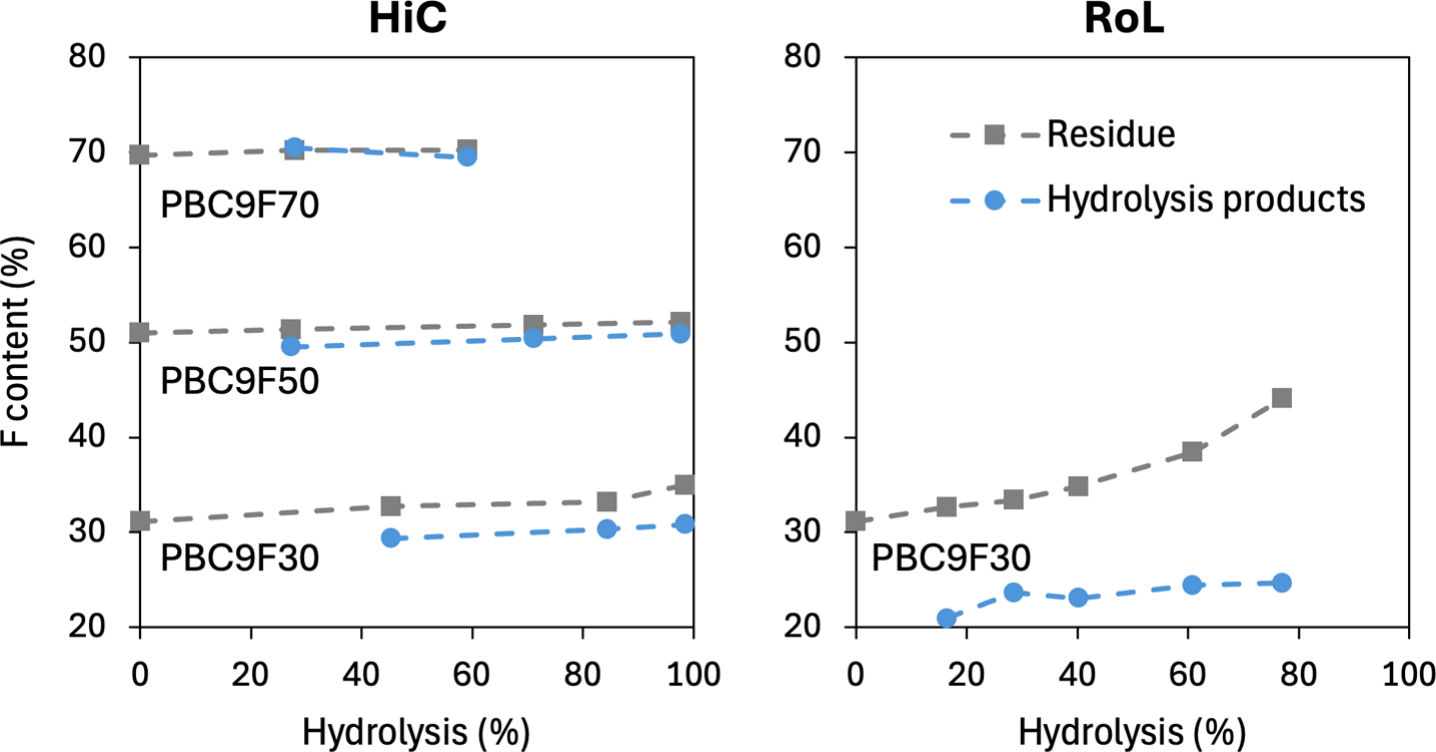
2,5-Furandicarboxylate (F) content (i.e., molar ratio of F relative to total diacids, determined by ^1^H NMR analysis) of both the solid polyester residues and the dissolved hydrolysis products during hydrolysis by *H. insolens* cutinase (HiC) (left) or *R. oryzae* lipase (RoL) (right), shown as a function of the polyester hydrolysis extent (%). The polyester PBC9F30 was tested in both HiC- and RoL-catalyzed hydrolysis, while PBC9F50 and PBC9F70 were additionally tested with HiC only. A pronounced increase in F content was observed in the residual polyester during RoL-catalyzed hydrolysis, whereas HiC-catalyzed hydrolysis resulted in little or no increase in F content.

### Implications

This work uses a series of biobased aliphatic–aromatic F- (and T-) containing copolyesters with anticipated biodegradability to establish a link between polyester chemical structure and enzymatic hydrolyzability, a critical—and often considered rate-determining—step in the biodegradation of these polyesters in many receiving environments.

For the design of F-containing biobased and biodegradable polyesters, this work demonstrates that the F content is the key structural variable controlling enzymatic hydrolyzability (and thus likely biodegradability). Material design thus needs to find a balance between the F content needed to ensure polyester performance during the application—with many demanding applications requiring higher F contents—with the F content to reach targeted hydrolyzability and biodegradability performances. For instance, applications with modest material performance requirements (e.g., short-lived items such as firework tips) and low F contents may suffice to ensure rapid enzymatic hydrolysis when released to the environment. By contrast, for more demanding applications, such as thin agricultural mulch films, higher F contents may be needed to ensure adequate mechanical properties during the processing and use phases; yet, they should not exceed critical threshold values above which enzymatic hydrolysis and biodegradation proceed only very slow. Importantly, this study shows that polyesters with F contents that are considerably higher than those of comparable T-based polyesters remain enzymatically hydrolyzable.

Although the effects of diol and aliphatic diacid chain lengths on hydrolyzability were less pronounced, their combined influence may be leveraged in material design to fine-tune biodegradability characteristics. Specifically, pairing the short-chain diol ethylene glycol with longer-chain aliphatic diacids (C9 or C10) enhanced the enzymatic hydrolyzability of polyesters across the entire range of tested F contents.

This work further postulates molecular-level explanations for the observed effects of polyester structure on enzymatic hydrolysis, which can be further tested in future work. The resulting fundamental understanding is critical to advance design principles for biobased F-containing polyesters with tunable biodegradability. Current effort is directed toward evaluating how soil biodegradability in laboratory incubations of the same set of polyesters tested herein correlates with their enzymatic hydrolyzability determined in this work. While the absolute rate of enzymatic hydrolysis and soil biodegradability will differ for any of the tested polyesters, this effort will establish the extent to which enzymatic hydrolyzability assays capture relative trends in soil biodegradability for a larger set of polyesters. These insights provide a foundation for implementing benign-by-design criteria—integrating hydrolyzability and, by extension, biodegradability—into the next-generation biobased and biodegradable polyesters, supporting the transition toward a sustainable and circular plastics economy.

## Supplementary Material



## Notes

Chemicals for copolyester synthesis, characterization of pure polyesters by ^1^H NMR, and additional figures, including correlation plots, ^1^H NMR spectra, enzymatic hydrolysis profiles, GPC elution profiles, and surface free energy measurements (PDF)
