# Supplementary material for: Biobased and Biodegradable Furandicarboxylate Polyesters: Linking Molecular Structure to Enzymatic Hydrolyzability
Source: Environ Sci Technol. 2026 Jun 22;60(26):18854–64. doi: 10.1021/acs.est.6c01921 (PMC13348168; doi:10.1021/acs.est.6c01921)
Supplement: Supplementary file 1 [file es6c01921_si_001.pdf]

Supporting Information

Bio-Based and Biodegradable Furandicarboxylate Polyesters: Linking Molecular Structure to Enzymatic Hydrolyzability

Thijs Vangeel[1], Yannick Matt[2], Lukas Becker[2], Kai Oliver Siegenthaler[3], Michael Sander[1,4*]

[1] Institute of Biogeochemistry and Pollutant Dynamics (IBP), Department of Environmental Systems Science, ETH Zurich, 8092 Zurich, Switzerland

[2] Polycondensation Research, RGR/BS, BASF SE, Carl-Bosch-Strasse 38, Ludwigshafen 67056, Germany

[3] Biodegradation & Microplastic Research, RGS/AB, BASF SE, Carl-Bosch-Strasse 38, Ludwigshafen 67056, Germany

[4] Department of Process Engineering, Swiss Federal Institute of Aquatic Science and Technology (Eawag), 8600 Dübendorf, Switzerland

*Corresponding author:

Michael Sander; email: michael.sander@env.ethz.ch

Section S1: Copolyester synthesis

Chemicals and solvents

Ethylene glycol (>98%) was purchased from Chemsolute. Sebacic acid and 1,3-propanediol (98%) were purchased from Thermo Scientific. Azelaic acid (98%) and 1,4-butanediol (99%) were purchased from abcr GmbH. Terephthalic acid (>98%) was purchased from Alfa Aesar. 2,5-Furandicarboxylic acid (polymer grade) was provided by Avantium. Succinic acid (>99%) and Tetrabutyl orthotitanate (>97%) were purchased from Merck.

Section S2: Characterization of pure polyesters

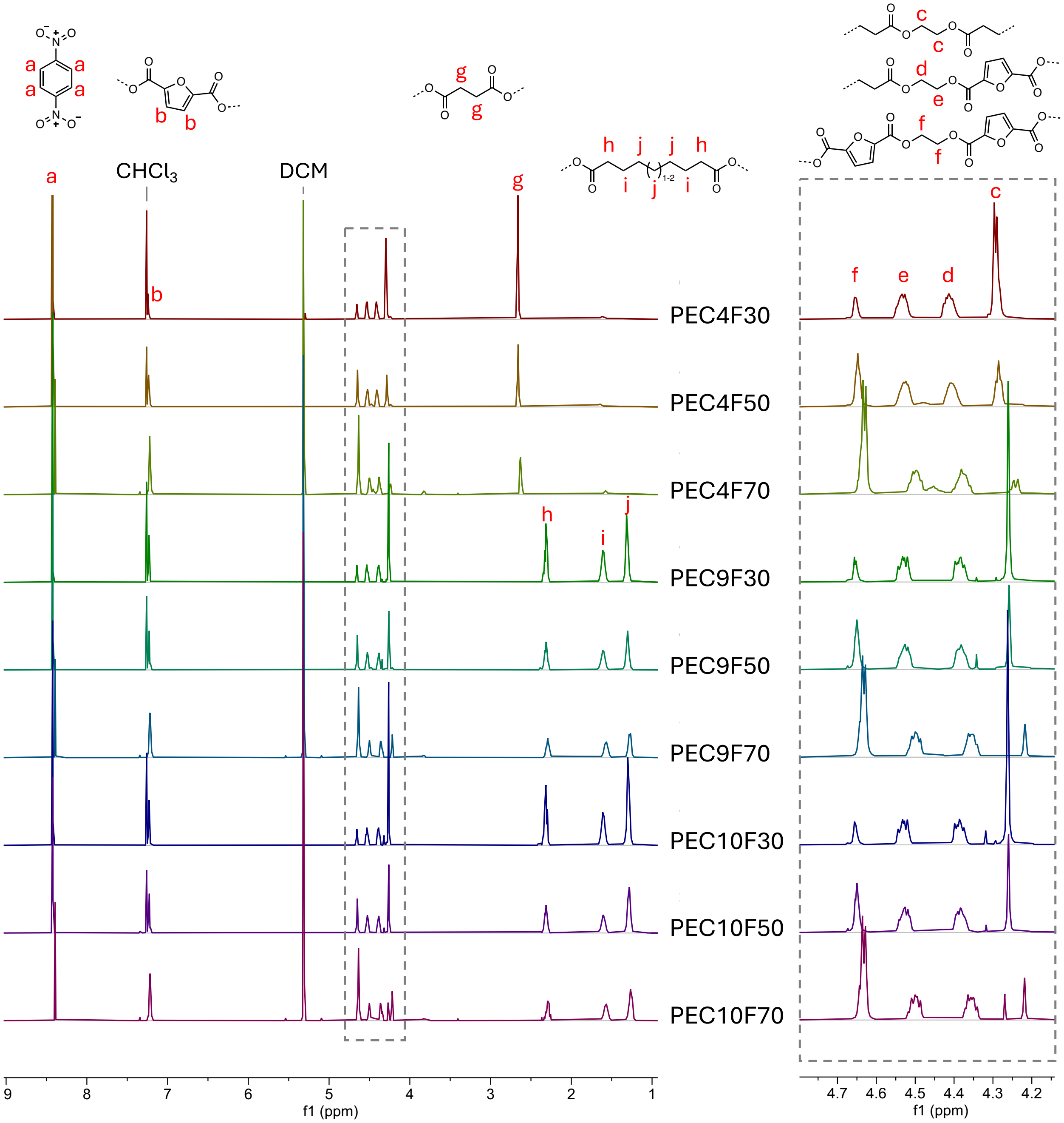

Figure S1: Annotated ¹H-NMR spectra of pure, F-containing polyesters synthesized with ethylene glycol as diol. Polyester names correspond to those listed in Table 1 of the main article. Note: PEC4F70, PEC9F70, and PEC10F70 were dissolved in deuterated dichloromethane instead of deuterated chloroform.

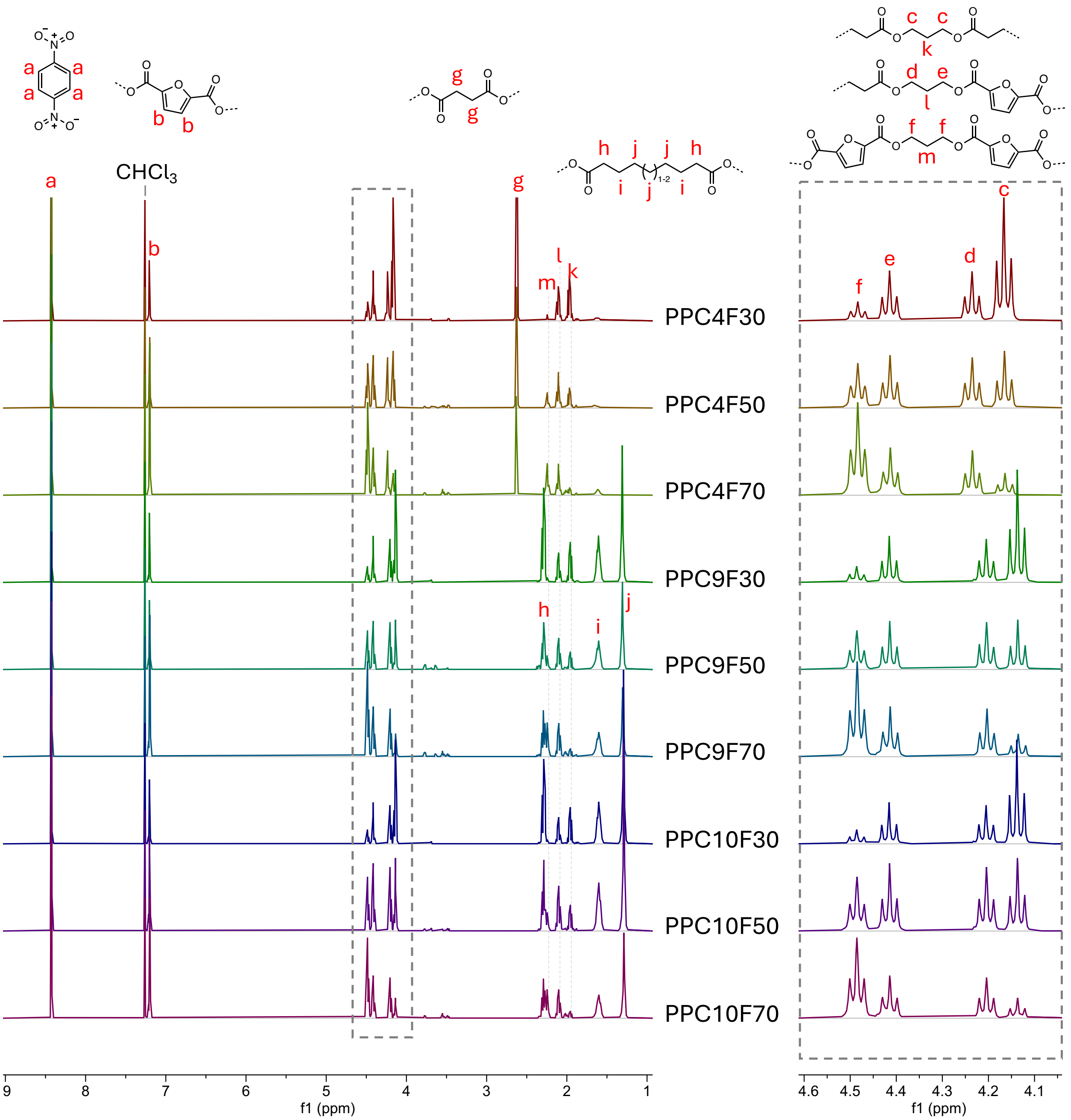

Figure S2: Annotated ^{1}H-NMR spectra of pure, F-containing polyesters synthesized with 1,3-propanediol as diol. Polyester names correspond to those listed in Table 1 of the main article.

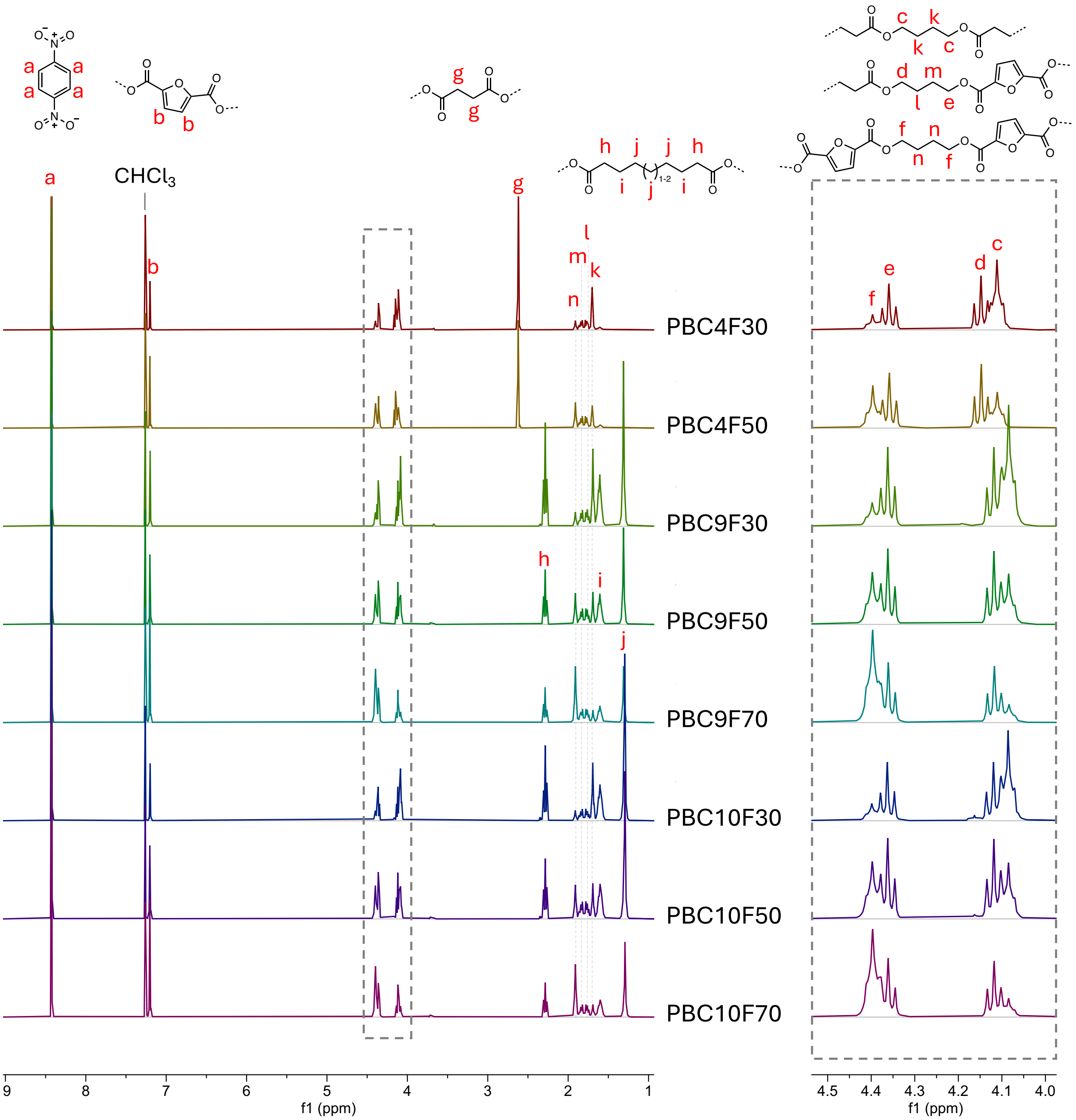

Figure S3: Annotated ^{1}H-NMR spectra of pure, F-containing polyesters synthesized with 1,4-butanediol as diol. Polyester names correspond to those listed in Table 1 of the main article.

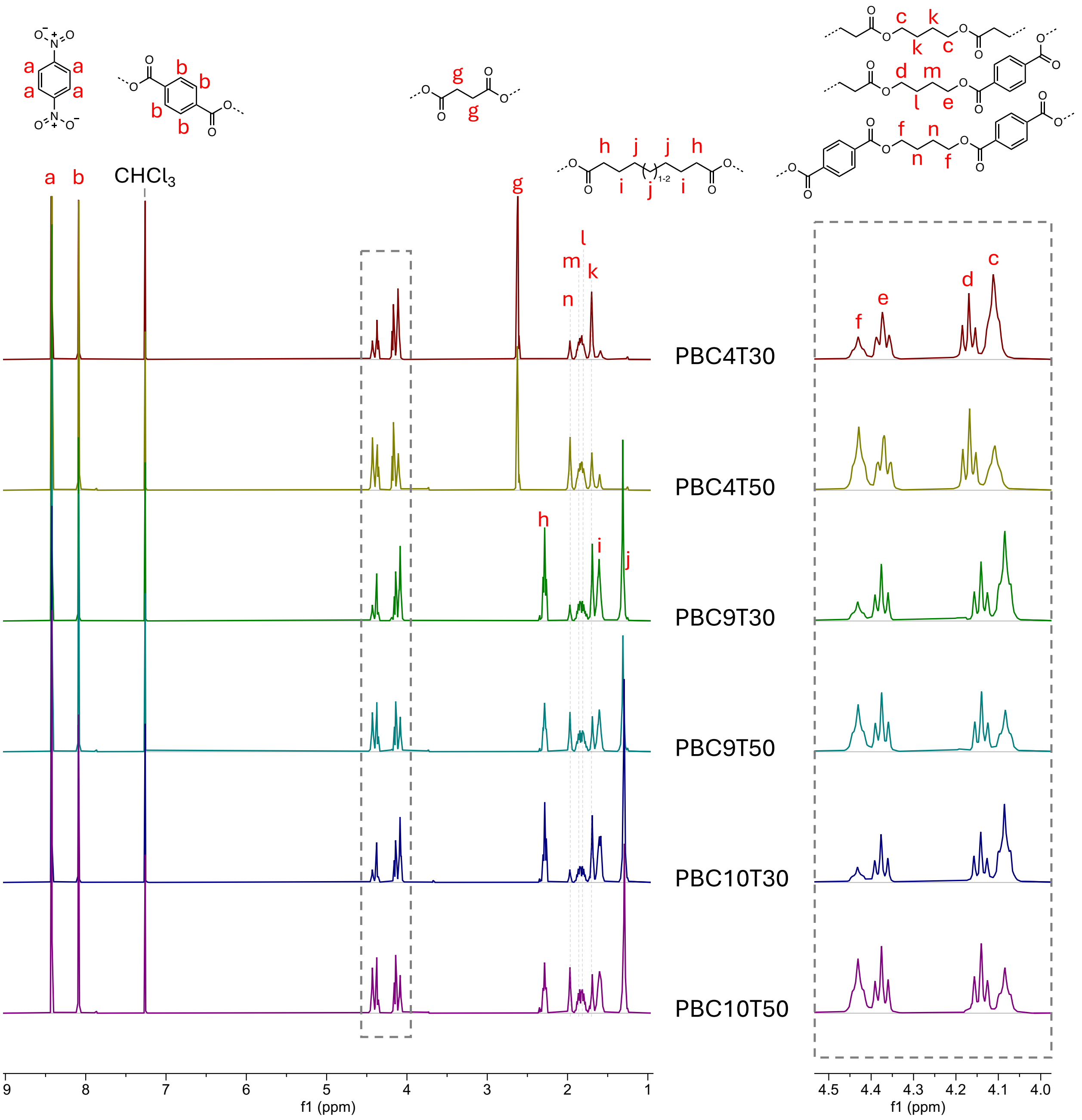

Figure S4: Annotated ^{1}H-NMR spectra of pure, T-containing polyesters synthesized with 1,4-butanediol as diol. Polyester names correspond to those listed in Table 1 of the main article.

Section S3: Results and Discussion – Additional Figures

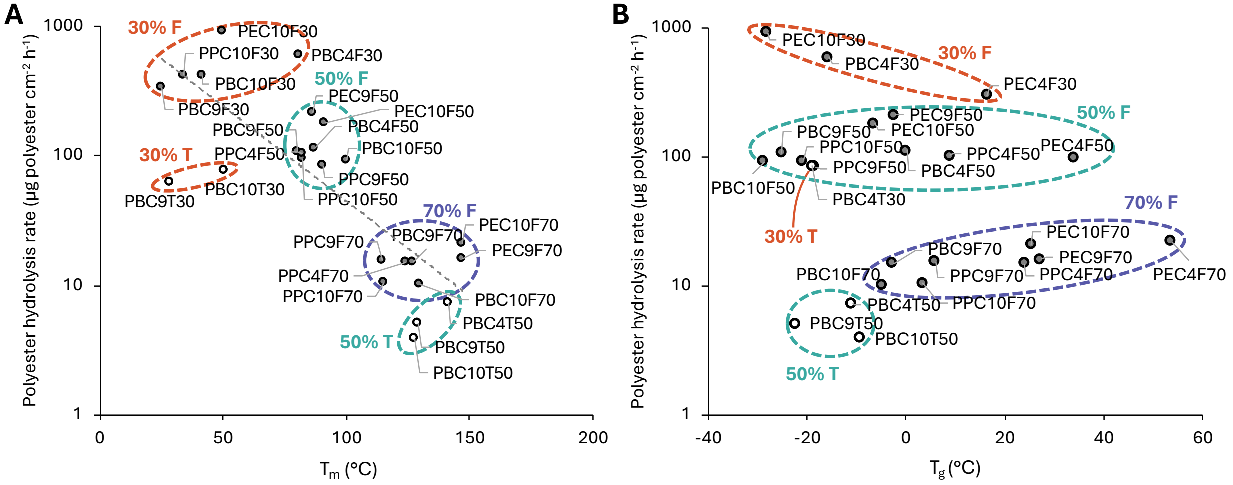

Figure S5: Correlation between HiC-catalyzed polyester hydrolysis rate and (A) polyester melting temperature (T_m) and (B) polyester glass transition temperature (T_g). Polyesters containing 2,5-furandicarboxylic acid (F) are shown as gray-filled dots, and those containing terephthalic acid (T) as white-filled dots. Dashed ellipses group polyesters with identical F or T contents. Polyesters with T_g < -30 °C are omitted from part B, as such low T_g could not be determined.

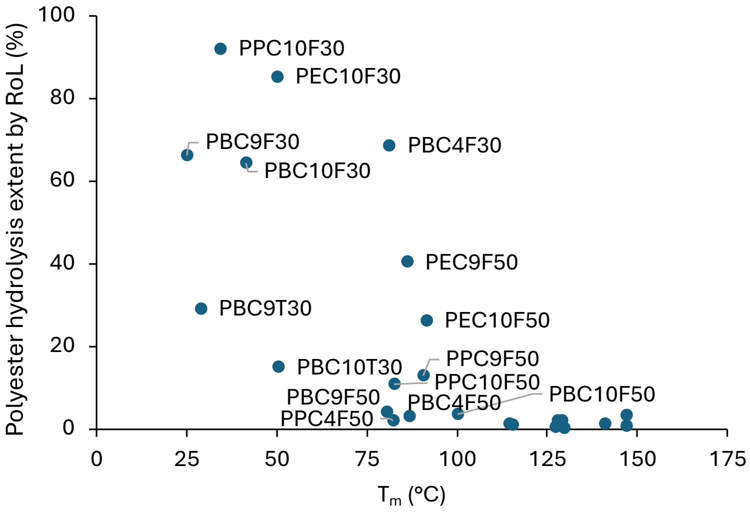

Figure S6: Correlation between polyester melting temperature and the extent of RoL-catalyzed polyester hydrolysis after 120h. Polyesters with a T_m higher than 100 °C (not labeled) are hardly hydrolyzed by RoL.

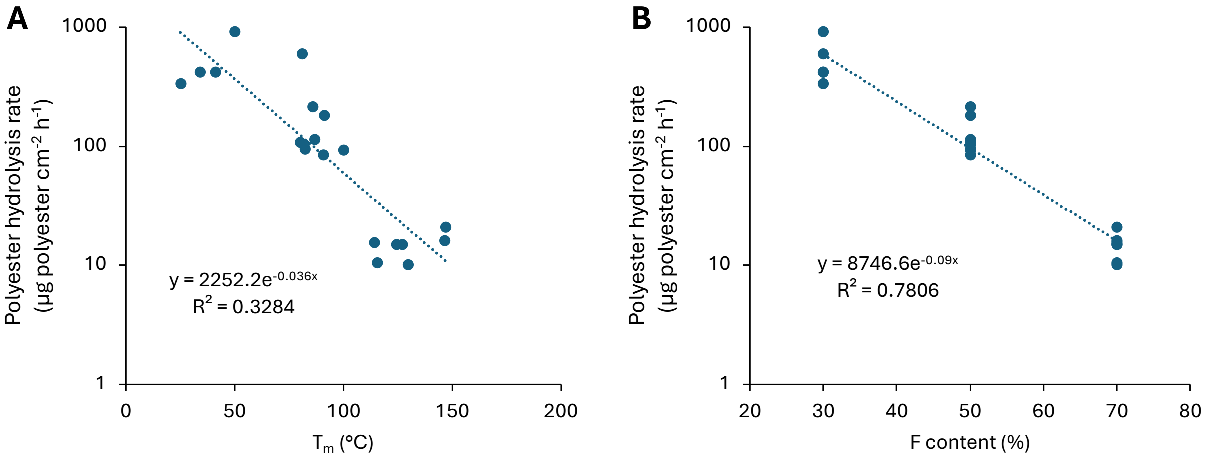

Figure S7: (A) Correlation between polyester melting temperature (T_m) and the polyester hydrolysis rate by HiC. (B) Correlation between F content and the polyester hydrolysis rate by HiC. In panel B, only those F-containing polyesters are included in the analysis that had a measurable melting temperature (i.e., the same ones as shown in panel A).

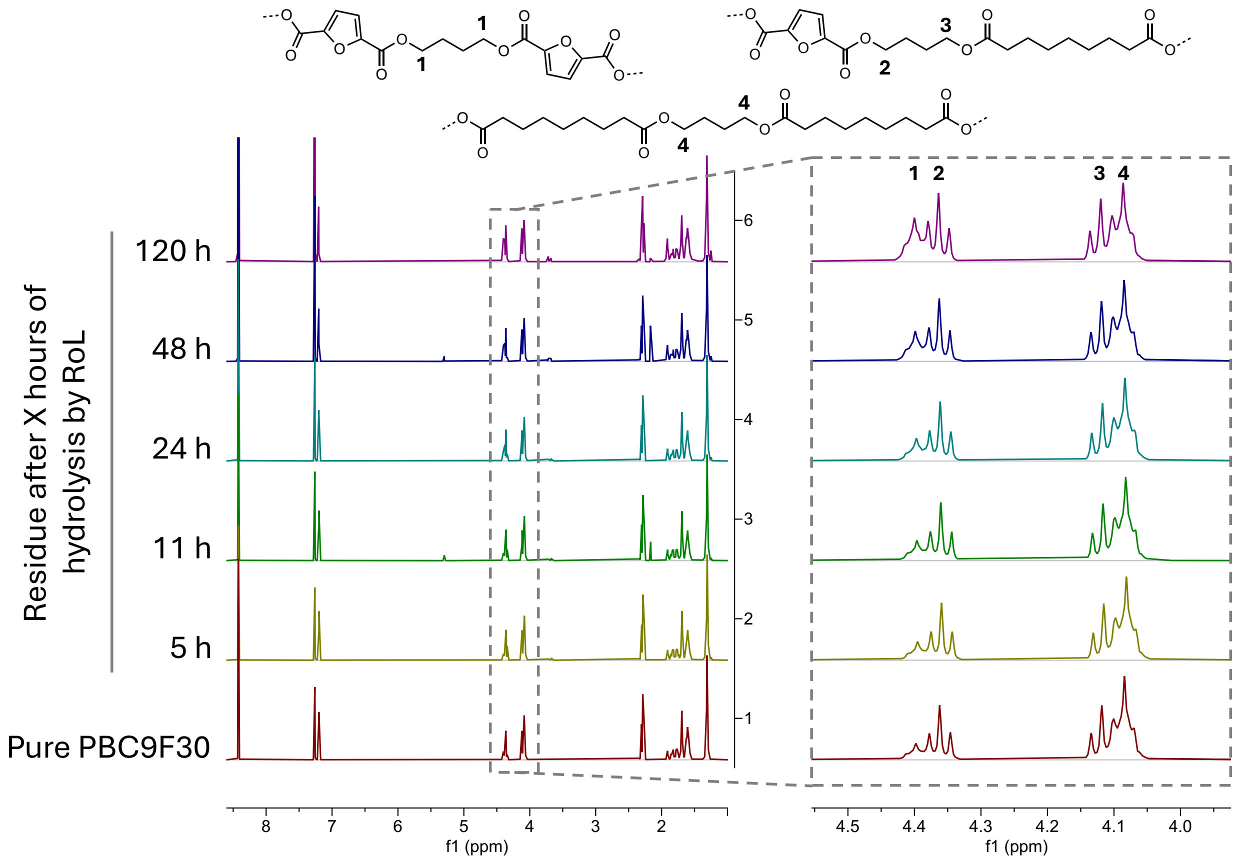

Figure S8: Annotated ^{1}H-NMR spectra of the polyester PBC9F30 (bottom) and its solid residues after hydrolysis by RoL for different times. The residue shows a progressive increase in F content over the course of the hydrolysis.

Table S1: Thermal properties of duplicate, solvent-cast polyester films, determined by Differential Scanning Calorimetry (DSC) (first heating scan). For PBC9F30, measuring the second heating scan was required as the standby temperature of the DSC (38 °C) was above its T_m. Thermal annealing was carried out on solvent-cast films at 70 °C for 2h. Thermal annealing resulted in increased ΔH_m for PBC9F50, indicating that these films had higher crystallinities.

Polyester	T_g [°C]	T_m [°C]	ΔH_m [J/g]
PBC9F30	-	26.6	16.1
PBC9F30	-	24.7	16.1
PBC9F30 annealed	-	24.9	18.4
PBC9F30 annealed	-	26.1	18.3
PBC9F50	-24.5	101.2	5.9
PBC9F50	-26.0	101.4	6.6
PBC9F50 annealed	-25.3	87.6	9.7
PBC9F50 annealed	-25.8	87.3	11.1
PBC9F70	-5.2	130.3	19.6
PBC9F70	-5.8	130.3	19.4
PBC9F70 annealed	-6.8	129.8	18.5
PBC9F70 annealed	-4.2	131.5	20.1

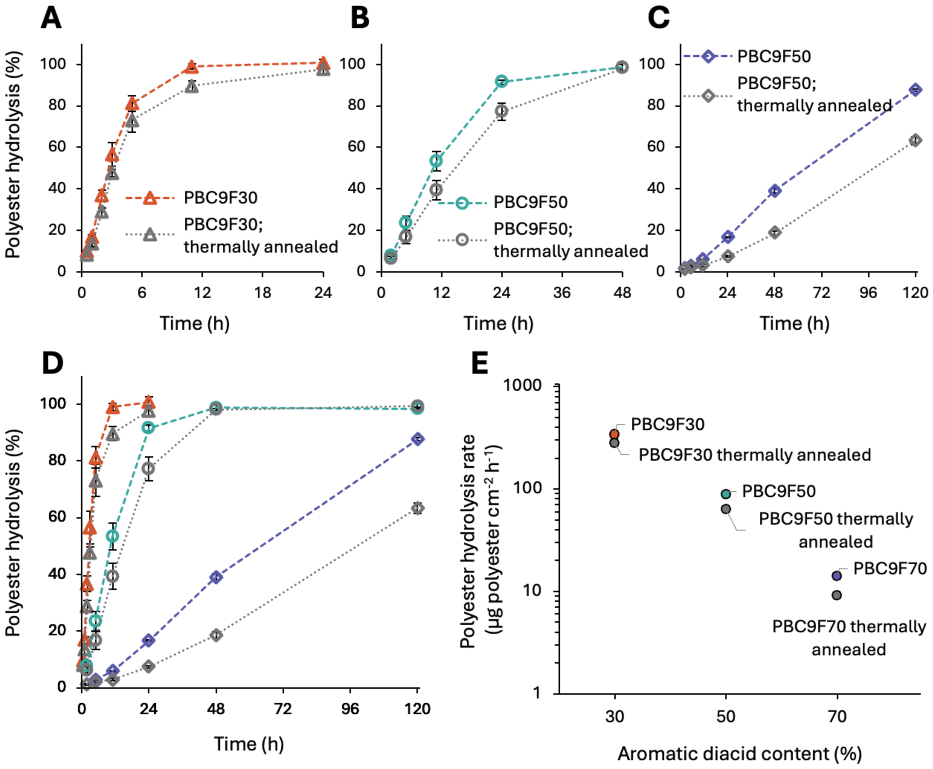

Figure S9: Enzymatic hydrolysis of thermally annealed polyester films (PBC9F30, PBC9F50 and PBC9F70), as compared to non-annealed analogues, by Humicola insolens cutinase (HiC) at pH 7.1 and 25 °C. Small products released during hydrolysis from polyester films into solution were quantified by dissolved total organic carbon (TOC) analysis. Symbols and error bars represent means and corresponding standard deviations of triplicate experiments for each polyester. (A-C) Comparison of regular solvent-cast films to films thermally annealed at 70 °C for 2h. (D) Combined figure showing the hydrolysis dynamics of (annealed) PBC9F30, PBC9F50, and PBC9F70. (E) Enzymatic hydrolysis rates of (thermally annealed) polyester films as a function of aromatic diacid content. Hydrolysis rates are calculated as the average surface-area-normalized rate up to 50% hydrolysis extent. These results illustrate that thermal annealing had a smaller effect on sample hydrolyzability as compared to polyester F content.

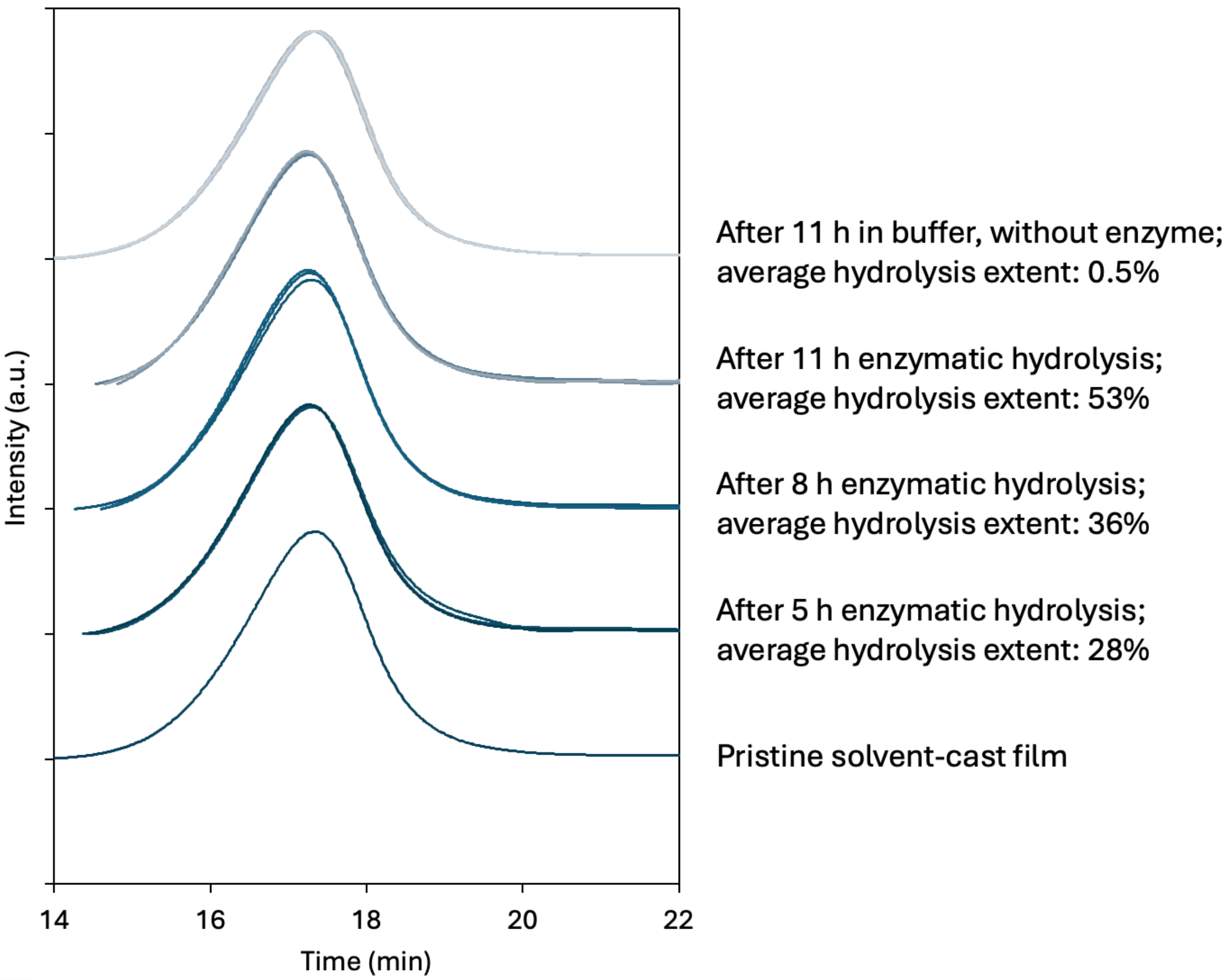

Figure S10: Gel permeation chromatography (GPC) elution profiles of PBC9F50 solvent-cast films. Area-normalized elution profiles are shown for the pristine solvent-cast film, the residual solid polyester after different extents of enzymatic hydrolysis (collected on a 0.45 µm PVDF filter), and the solid polyester film after incubation in buffer only (i.e., no enzyme control). Enzymatic hydrolysis was performed using Humicola insolens cutinase (HiC) at pH 7.1 and 25°C. Each elution profile shows data from triplicate hydrolysis incubations. The overlap in the elution profiles demonstratse that the molecular weight distribution of the bulk polyester remained unchanged during enzymatic polyester hydrolysis, consistent with enzymatic hydrolysis occurring at the polyester surface while the polyester bulk was not affected.

Table S2: Estimated surface free energies (SFEs) for solvent-cast polyester films of the PBC9Fx and PBC9Tx series. Total SFE and its dispersive and polar components were derived from contact angle measurements using water, diiodomethane, and formamide. All polyesters exhibited similar total SFE values (~ 39–44 mN/m) with a dominant dispersive component, regardless of the aromatic diacid type or content. The marginal variations in SFE (and its low polar component) observed do not correlate with the large differences in enzymatic hydrolysis rates of these films, indicating that surface hydrophobicity is not a main determinant for hydrolysis of these polyesters by Humicola insolens cutinase (HiC).

	**Contact Angle (°)**			**Surface Energy (mN/m)**			**Fit quality**
Sample	Water	Diiodomethane	Formamide	Dispersive	Polar	Total	R^2
PBC9F30	87 ± 5	33 ± 3	61 ± 4	43 ± 2	1 ± 1	44 ± 2	0.93
PBC9F50	93 ± 2	36 ± 3	60 ± 1	42 ± 0	0 ± 0	42 ± 1	1
PBC9F70	82 ± 3	47 ± 0	58 ± 1	35 ± 2	3 ± 1	39 ± 2	1
PBC9T30	85 ± 1	36 ± 3	59 ± 4	40 ± 3	2 ± 1	42 ± 4	0.98
PBC9T50	89 ± 5	40 ± 3	62 ± 5	38 ± 2	1 ± 0	40 ± 3	0.99
